# Angelica sinensis polysaccharide (ASP) attenuates diosbulbin-B (DB)-induced hepatotoxicity through activating the MEK/ERK pathway

**DOI:** 10.1080/21655979.2021.1950280

**Published:** 2021-07-06

**Authors:** Chunfeng Li, Shumin Liu, Jian Zheng, Yingwei Xue

**Affiliations:** a Department of Gastrointestinal Surgical Ward, Harbin Medical University Cancer Hospital, Harbin, Heilongjiang, China; b Institute of Chinese Medicine, Heilongjiang University of Chinese Medicine, Harbin, Heilongjiang, China; c Department of Diagnostic Radiology Division, Harbin Medical University Cancer Hospital, Harbin, Heilongjiang, China

**Keywords:** *Angelica sinensis* polysaccharide, diosbulbin-B, hepatotoxicity, autophagy, apoptosis, MEK/ERK pathway

## Abstract

Diosbulbin-B (DB) is a promising therapeutic drug for cancer treatment; however, DB-induced hepatotoxicity seriously limits its clinical utilization. Based on this, the present study investigated whether the *Angelica sinensis* extract, angelica sinensis polysaccharide (ASP), was effective to attenuate DB-induced cytotoxicity in hepatocytes. The primary hepatocytes were isolated from rats and cultured *in vitro*, which were subsequently treated with high-dose DB (100 μM) and ASP (12 μg/ml) to establish the DB-induced hepatotoxicity models. MTT assay and flow cytometry (FCM) were performed to evaluate cell viability, and the results showed that high-dose DB-induced cell apoptosis and inhibition of proliferation were reversed by co-treating cells with ASP, which were supported by our Western Blot assay data that ASP upregulated Cyclin D1 and CDK2 to abrogate high-dose DB-induced cell cycle arrest. In addition, ASP exerted its regulating effects on cell autophagy, and we found that ASP increased LC3B-II/I ratio and Atg5, but decreased p62 to activate the autophagy flux. Of note, the MEK/ERK pathway could be activated by ASP in the DB-treated hepatocytes, and the protective effects of ASP on high-dose DB-induced hepatocyte death were abolished by co-treating cells with the autophagy inhibitor (3-methyladenine, 3-MA) and MEK/ERK selective inhibitor (SCH772984). Moreover, blockage of the MEK/ERK pathway suppressed cell autophagy in the hepatocytes co-treated with ASP and high-dose DB. Taken together, this *in vitro* study illustrated that ASP activated the MEK/ERK pathway mediated autophagy to suppress high-dose DB-induced hepatotoxicity.

## abstract graphical

**Figure:**
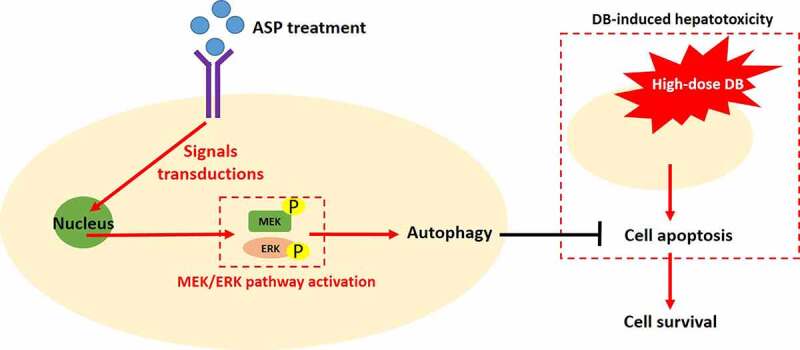

## Introduction

Diosbulbin-B (DB) is a promising anti-tumor ingredient which is extracted from Chinese medicine *Dioscorea bulbifera L* [1–3], and the underlying mechanisms by which DB suppresses cancer progression and increases cisplatin-sensitivity in gastric cancer (GC) have been preliminarily investigated and discussed in our previous publications [4,5]. Currently, the main problem that blocks DB’s clinical utilization is DB-induced hepatotoxicity [6–8]. Our published data indicate that low-dose DB specifically induces cell death in GC cells but not in the normal hepatocytes, and targeting the associated tumor-associated genes combined with low-dose DB co-treatments are effective to hamper GC progression [4,5]. Specifically, knockdown of circRNA CDR1as specifically triggered low-dose DB-induced cell death in GC [4], and targeting programmed death ligand-1 (PD-L1) combined with low-dose DB also increased the susceptibility of GC cells to cisplatin treatment [5]. However, the cytotoxic effects of low-dose DB alone on cancer cells are largely limited, which made utilization of high-dose DB for cancer treatment become necessary. Thus, we designed this study to search for the alternative treatment strategies or agents that are capable of ameliorating high-dose DB induced hepatotoxicity, which may help to maximize the tumor-suppressing effects of single high-dose DB treatment for clinical utilization.

Angelica sinensis polysaccharide (ASP) is an active constituent extracted from a water extract of *Angelica sinensis* [9–13], which has been used for the treatments of various diseases, including ethylene glycol-induced calcium oxalate urolithiasis [14], aplastic anemia [15], liver injury [16]. Interestingly, ASP has been validated as an ideal and effective therapy drug for liver injury [16–18], specifically, Cao et al. report that ASP suppresses oxidative stress and hepatic apoptosis to attenuate acetaminophen-induced liver injury [16], Wang et al. find that ASP regulates the IL-22/STAT3 pathway to alleviate CCl(4)-induced liver fibrosis [18], and Gao et al. support that ASP ameliorates hepatic injury in mice models [17]. In addition, interplay of autophagy and apoptosis plays important role in regulating the development of liver injury [19–21], and to our knowledge, induction of protective autophagy suppressed cell apoptosis [22–24], and DB promotes liver injury by inducing apoptotic cell death [4,2^5^, 5] and inactivation of autophagy flux [25]. Of note, ASP is capable of regulating both cell autophagy [26] and apoptosis [15,16], but it is still largely unknown whether ASP can attenuate DB-induced hepatotoxicity via modulating cell autophagy and apoptosis.

The MEK/ERK signaling pathway is closely associated with liver injury [27,28], which involves in regulating both cell apoptosis [29,30] and autophagy [31]. For example, Fu et al. find that activation of the MEK/ERK pathway ameliorates hypoxic/ischemic-induced bone mesenchymal stem cell apoptosis [29], and Wang et al. evidence that MEK/ERK-induced autophagy contributes to cell survival in IPEC-J2 cells with Ochratoxin A exposure [31]. Notably, according to the data from Xu et al., ASP regulates the MEK/ERK pathway to induce protective autophagy and suppress sodium nitroprusside (SNP)-induced cell apoptosis in osteoarthritis chondrocytes [26], which convinces us to speculate that ASP may regulate the MEK/ERK pathway mediated autophagy to attenuate DB-induced cell apoptosis in hepatocytes.

Based on the published data, we hypothesized that ASP might be an ideal candidate agent to attenuate high-dose DB-induced hepatotoxicity, and the cell autophagy and apoptosis might be critical for this process. Thus, we performed the present study to explore the alleviating effects of ASP on high-dose DB-induced cytotoxicity in rat hepatocytes, and uncover the potential underlying mechanisms by which ASP exerted its protective effects.

## Materials and methods

### Cell isolation, culture and treatment

The primary rat hepatocytes were isolated, purified, and cultured *in vitro* according to the experimental protocols provided by the previous study [16]. Briefly, the rats were purchased from the Research Animal Center of Harbin Medical University, which were anesthetized, sterilized and dissected in germ-free laboratory. The rat liver was dissected and the desmosomes were disrupted by using the Ca ^2+^ free buffer solution, and the hepatocytes were obtained by incubating the liver tissues with Ca ^2+^ containing collagenase buffer. The cells were then purified and cultured in the collagen-coated 6-well plates overnight in the Williams’ E medium (ThermoFisher Scientific, USA) in the standard culture conditions with 5% CO_2_ at 37 °C for further utilization. All the animal experiments were approved by the Ethics Committee of Harbin Medical University Cancer Hospital. Finally, the cells were respectively treated with high-dose DB (100 μM), ASP (12 μg/ml), 3-MA and SCH772984 as previously described [16].

## Western blot analysis

As previously described [16], total proteins were extracted from the rat hepatocytes by using the RIPA lysis buffer (Beyotime, Shanghai, China) in keeping with the manufacturer’s protocols. The concentrations of total proteins were quantified by BCA kit (ThermoFisher Scientific, USA) and were separated by performing 12% SDS-PAGE. The target proteins were then transferred onto the PVDF membranes, which were blocked by 5% skimmed milk and were incubated with primary antibodies against Cyclin D1 (1:2000), CDK2 (1:1500), LC3B (1:1500), Atg5 (1:2000), p62 (1:2000) and GAPDH (1:2000) purchased from Cell Signaling Technology (USA) at 4 °C overnight, which were subsequently washed by TBST buffer and the membranes were incubated with the secondary antibody (1:4000, Cell Signaling Technology, USA) for 1 h at room temperature. Finally, the ECL system (ThermoFisher Scientific, USA) was used to visualize the protein bands, which were analyzed by performing the Image J software.

## 3-(4,5-Dimethylthiazol-2-yl)-2,5-diphenyltetrazolium bromide (MTT) assay

The rat hepatocytes were seeded onto the 96-well plates at the density of 1 × 10^5^ cells per well, which were subsequently subjected to ASP and DB treatment for 0 h, 6 h, 12 h, 18 h and 24 h in the incubator, and the MTT reaction solution was added in the 96-well plates with 20 μl per well for 4 h incubation. Then, the supernatants were removed and the blue formazan was dissolved by using the DMSO solution with 150 μl per well, and the plates were fully vortexed and a microplate reader (ThermoFisher Scientific, USA) was used to examine the optical density (OD) in each well, which could represent the relative cell proliferation abilities in the hepatocytes.

## Flow cytometry (FCM)

To examine cell apoptosis ratio, the hepatocytes were pre-subjected to different treatments and incubated with Annexin V-FITC (BD Biosciences, USA) and propidium (BD Biosciences, USA) for 30 min at room temperature without light exposure. A FACSCalibur cytometer (BD Biosciences, USA) was performed to examine the Annexin V-FITC and PI-positive apoptotic cells. The raw data were obtained and analyzed by using the FlowJo 10 software (Tree Star, USA).

## Data analysis and visualization

The data were analyzed by using the SPSS 18.0 software, and were visualized by using the GraphPad Prism 8.0 software. We represented the data as means ± standard deviation (SD), and means from two groups were compared by using the Student’s t-test, and one-way ANOVA analysis was conducted to analyze the significance of the means from different groups. *P* < 0.05 was considered as statistical significance and was marked by ‘*’.

## Results

### ASP rescued cell proliferation and viability in DB-treated hepatocytes

**Figure 1.**
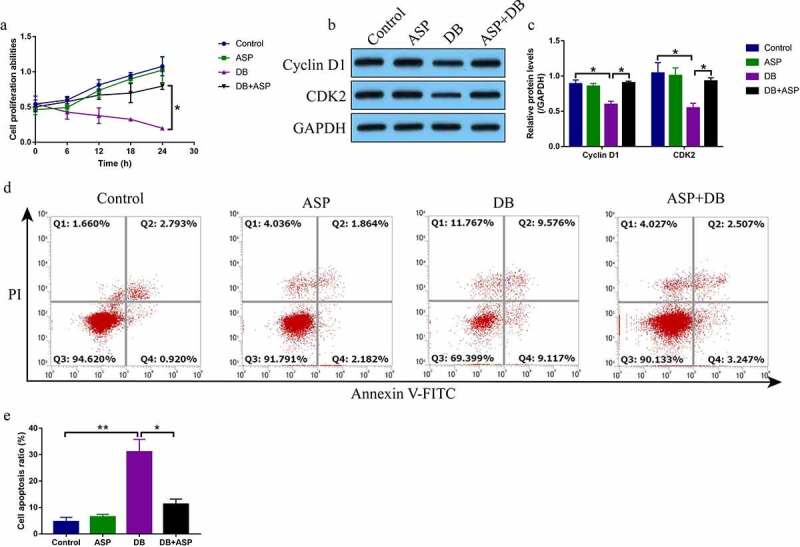
DB-induced cell apoptosis was reversed by ASP co-treatment. (a) MTT assay was used to examine cell proliferation, and (b, c) Western Blot analysis was performed to determine Cyclin D1 and CDK2 expressions. (d, e) Cell apoptosis ratio was measured by performing FCM assay through staining cells with Annexin V-FITC and PI. Each experiment had 3 repetitions, and * *P* < 0.05

According to the preliminary data from our previous publications [4,5] and other teams [16,26] that high-dose DB exerted its cytotoxic effects on hepatocytes and ASP is effective to attenuate liver injury, we explored whether ASP attenuated high-dose DB-induced cell death in the hepatocytes. The primary rat hepatocytes were sequentially exposed to high-dose DB (100 μM) and ASP (12 μg/ml) according to our preliminary experiments (data not shown) and published data [4,5,16,26]. The MTT assay was used to determine cell proliferation abilities, and data in the Figure 1a showed that DB significantly suppressed cell proliferation in a time-dependent manner, while ASP alone had little effects on hepatocytes proliferation. Interestingly, the inhibiting effects of high-dose DB on cell proliferation in the hepatocytes were reversed by co-treating cells with ASP (Figure 1a). Also, by performing the Western Blot analysis, we proved that DB suppressed Cyclin D1 and CDK2 expressions to induce cell cycle arrest, which were also reversed by ASP co-treatment (Figure 1b,c). Moreover, the FCM results showed that ASP decreased cell apoptosis ratio in the hepatocytes co-treated with DB (Figure 1d,e).

## ASP triggered autophagy to protect hepatocytes from DB-induced cell death

**Figure 2.**
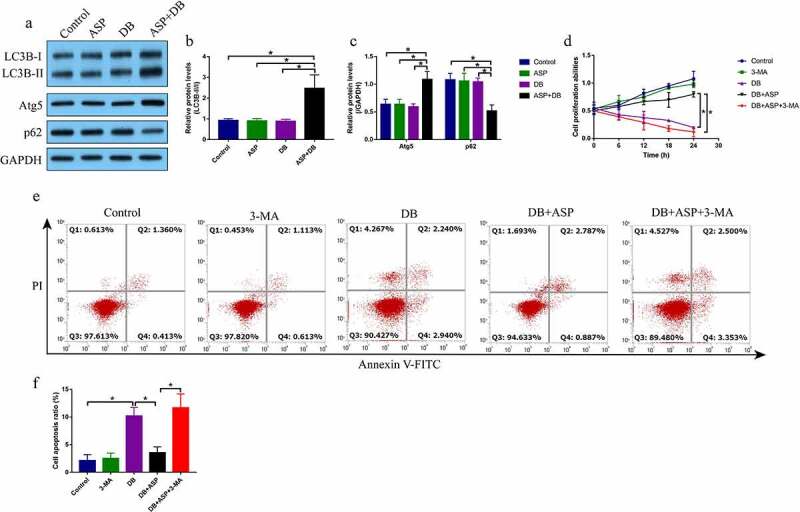
The autophagy flux was activated by ASP in DB-treated hepatocytes. (a-c) The autophagy associated biomarkers were examined by Western Blot analysis. (d) MTT assay was used for cell proliferation evaluation. (e, f) Cell apoptosis was examined by FCM assay. Each experiment had 3 repetitions, and * *P* < 0.05

Given that ASP played an important role in regulating cell autophagy [26], and there existed interplay between autophagy and apoptosis [22–24], we conjectured that ASP might regulate autophagy to reverse DB-induced hepatocyte death. To validate this hypothesis, the hepatocytes were treated with ASP and DB for 6 h according to our preliminary experiments (data not shown). By performing the Western Blot analysis, we evidenced that DB alone or ASP alone did not influence the autophagy flux, while ASP and DB co-treatment significantly increased the levels of LC3B-II/I ratio and Atg5, but promoted p62 degradation in the hepatocytes (Figure 2a-c). Next, the hepatocytes were treated with ASP, DB and autophagy inhibitor 3-MA, as shown in Figure 2d, the promoting effects of ASP on cell proliferation in DB-treated hepatocytes were abrogated by co-treating cells with 3-MA. Similarly, 3-MA promoted cell apoptosis in the ASP and DB co-treated hepatocytes (Figure 2e,f), indicating that ASP exerted its protective effects on DB-induced hepatocyte death via inducing autophagy.

## ASP promoted autophagic flux in DB-treated hepatocytes via activating the MEK/ERK pathway

**Figure 3.**
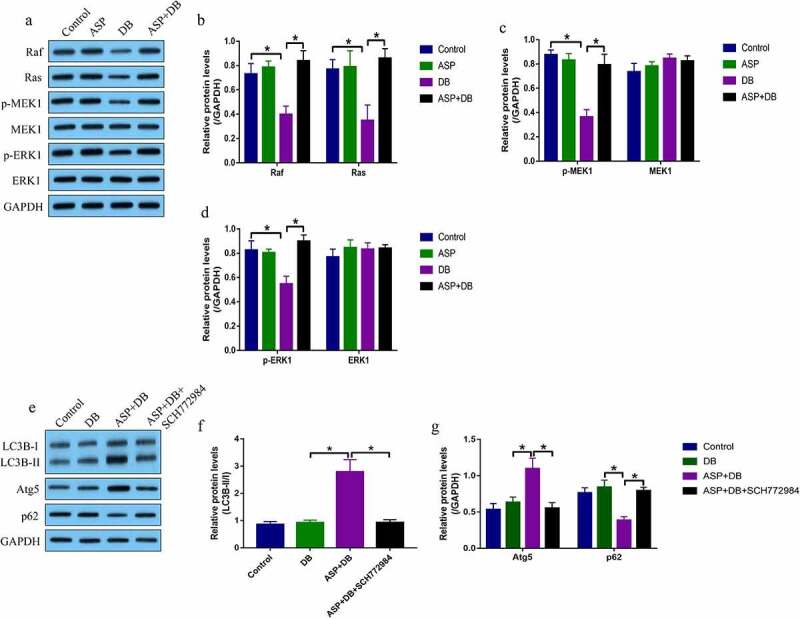
ASP activated the MEK/ERK pathway to trigger cell autophagy. We performed western blot analysis to examine the expression status of (a-d) Raf, Ras, p-MEK1, MEK1, p-ERK1 and ERK1, and (e-g) LC3BII/I ratio, Atg5 and p62 in the hepatocytes. Each experiment had 3 repetitions, and * *P* < 0.05

Next, we investigated the molecular mechanisms by which ASP regulated cell autophagy in the hepatocytes. As previously described, the MEK/ERK pathway was closely associated with cell autophagy, and this pathway mediated cell autophagy could be activated by ASP treatment in osteoarthritis chondrocytes [26], which enlightened us to investigate whether ASP regulated autophagy in hepatocytes via activating the MEK/ERK pathway. As shown in Figure 3a-d, by performing Western Blot analysis, we found that DB decreased the expression levels of Ras, Raf, phosphorylated MEK1 (p-MEK1), and phosphorylated ERK1 (p-ERK1) to inactivate the MEK/ERK pathway in the hepatocytes, which were reactivated by co-treating cells with ASP. Of note, ASP alone did not influence the MEK/ERK pathway (Figure 3a- d). Next, the hepatocytes were pre-treated with MEK/ERK selective inhibitor SCH772984, and the results showed that SCH772984 decreased LC3B-II/I ratio and Atg5, but increased p62 levels to suppress DB/ASP-induced autophagy in the hepatocytes (Figure 3e, f), suggesting that ASP activated the MEK/ERK pathway to trigger cell autophagy in the DB-treated hepatocytes.

## SCH772984 abrogated the protective effects of ASP on DB-induced hepatocyte death

**Figure 4.**
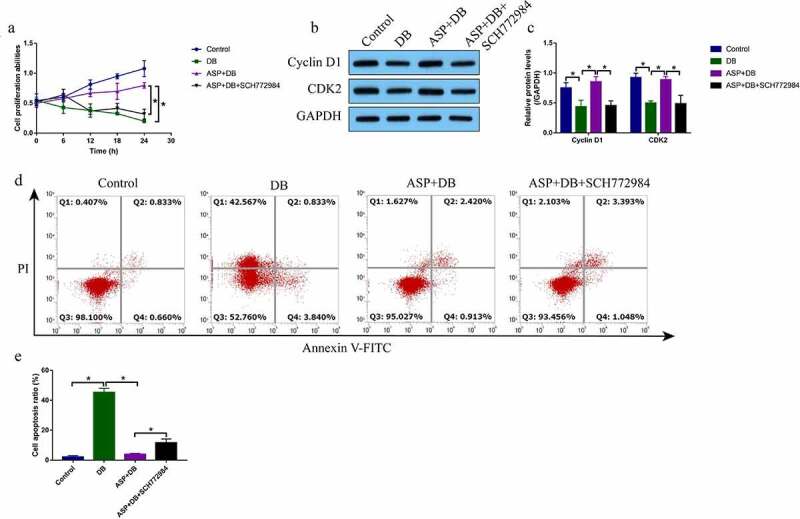
ASP regulated the MEK/ERK pathway to exerted its protective effects in DB treated hepatocytes. (a) SCH772984 suppressed cell proliferation in ASP and DB co-treated hepatocytes, as determined by MTT assay. (b, c) The expression levels of cyclin D1 and CDK2 were determined by western blot analysis. (d, e) FCM assay was used to evaluate cell apoptosis ratio in hepatocytes. each experiment had 3 repetitions, and * *P* < 0.05

Previous data showed that there existed interplays between cell autophagy and death, and activation of protective autophagy was effective to restore cell viability [22–24]. Since we had proved that ASP regulated autophagy in the hepatocytes via activating the MEK/ERK pathway, we conjectured that blockage of MEK/ERK pathway was capable of reversing the protective effects of ASP on DB-induced cell death. As shown in Figure 4a, the MTT assay results showed that ASP rescued cell proliferation abilities in the DB-treated hepatocytes, which were abrogated by co-treating cells with SCH772984. Also, the Western Blot analysis results supported that SCH772984 decreased the expression levels of Cyclin D1 and CDK2 to induce cell cycle arrest in DB/ASP co-treated hepatocytes (Figure 4b, c). In addition, the protective effects of ASP on DB-induced cell apoptosis were also abolished by SCH772984 (Figure 4d, e), implying that ASP rescued cell viability in DB treated hepatocytes via modulating the MEK/ERK pathway.

## Discussion

Diosbulbin-B (DB)-induced hepatotoxicity seriously limits the therapeutic efficacy of this Chinese medicine for clinical utilization [6–8], although our previous work has validated that low-dose DB combined with gene therapies specifically kills cancer cells instead of normal hepatocytes [4,5], the cytotoxic effects of low-dose DB alone on cancer cells are largely limited as the results of its low concentrations, which made utilization of high-dose DB on the treatment of cancers become necessary. Thus, it is reasonable and urgent to seek for adjuvant treatment strategies to ameliorate high-dose DB-induced cytotoxicity in hepatocytes, which helps to maximize DB’s therapeutic effects for cancer treatment. To our knowledge, DB induces cell apoptosis and inhibits cell viability in hepatocytes to promote liver injury [32,33], and inhibition of DB-induced apoptotic cell death is an effective strategy to attenuate DB-induced liver injury [4,2^5^, 5], which convinces us that identification of novel drugs that target cell apoptosis may help to solve this problem. According to the previous information that angelica sinensis polysaccharide (ASP) has been used for the treatment of liver injury by suppressing apoptotic cell death [16–18], we selected ASP for further investigations and evidenced that ASP was effective to rescued cell viability and suppressed DB-induced cell apoptosis in hepatocytes, suggesting that ASP was effective to reverse DB-induced cytotoxicity in hepatocytes, which were partially supported by the previous publications [16–18].

To our knowledge, interplays between cell autophagy and apoptosis participate in the regulation of various diseases, including liver injury [19–21], and previous literatures reported that induction of protective autophagy suppresses cell apoptosis to ameliorate liver injury and fibrosis [22–24]. In addition, cell autophagy can be modulated by various Chinese medicines, including ASP [26]. For example, Xu et al. report that ASP attenuates SNP-induced apoptosis in osteoarthritis chondrocytes via inducing protective autophagy [26]. Interestingly, DB also regulates cell autophagy, and Ye et al. evidence that DB induces mitochondria-dependent apoptosis in L-02 hepatocytes via modulating reactive oxygen species-mediated autophagy [25], which rendered the possibility that ASP might attenuate DB-induced cell apoptosis in hepatocytes via promoting autophagy. By performing the following experiments, we validated that ASP induced protective autophagy in DB-treated hepatocytes, and inhibition of cell autophagy by 3-MA promoted cell death in ASP and DB co-treated hepatocytes, suggesting that ASP rescued cell viability and inhibited cell apoptosis in hepatocytes by inducing autophagy.

The MEK/ERK pathway participates in the regulation of liver injury [27,28] by modulating both cell apoptosis [29] and autophagy [31], and interestingly, researchers notice that ASP regulates the MEK/ERK pathway in osteoarthritis chondrocytes [26], which were validated by our data that DB inactivated the MEK/ERK pathway in the chondrocytes, which were reactivated by co-treating cells with ASP, suggesting that ASP activated the MEK/ERK pathway in DB-treated hepatocytes. In addition, based on the published data [26], we evidenced that ASP promoted cell autophagy in DB-treated hepatocytes by activating the MEK/ERK pathway. In addition, we evidenced that inhibition of the MEK/ERK pathway suppressed cell viability and promoted cell apoptosis in the hepatocytes co-treated with ASP and DB, suggesting that ASP rescued cell functions in DB treated hepatocytes by activating the MEK/ERK pathway.

## Conclusions

In general, we proved that ASP attenuated DB-induced hepatotoxicity *in vitro*, and the potential underlying mechanisms were also uncovered. Mechanistically, ASP activated MEK/ERK pathway mediated autophagy to ameliorate DB-induced cell apoptosis in the hepatocytes. Our data supported that ASP could be used as adjuvant agent to combine with DB for cancer treatment in clinic. However, our preliminary results were still needed to be validated by the following *in vivo* animal experiments and the associated clinical data in our future work.
